# Sinogenic intracranial complications in children - cases report

**DOI:** 10.1186/1471-2334-14-S7-P63

**Published:** 2014-10-15

**Authors:** Monica Luminos, Anca Cristina Drăgănescu, Angelica Constanța Vişan, Magdalena Vasile, Mădălina Maria Merişescu, Cristina Negulescu, Gheorghiță Jugulete, Diana Maria Slavu, Cornelia Dogaru, Endis Osman, Anuța Bilaşco

**Affiliations:** 1 National Institute for Infectious Diseases "Prof. Dr. Matei Balş", Bucharest, Romania; 2 Carol Davila University of Medicine and Pharmacy, Bucharest, Romania

## Background

Although uncommon, intracranial suppurative complication of sinusitis in children can occur. The progressive pneumatization of the sinuses after birth and the late appearance of the sphenoid and frontal sinuses explain the predilection for intracranial sinogenic complications in older children and adolescents. Early imaging is crucial to diagnosis and brain MRI is the most useful test. Medical therapy combined with neurosurgical and otolaryngological surgical intervention may improve outcome and reduce the neurological sequelae.

## Case report

We present the cases of 2 girls admitted in the ICU of the National Institute for Infectious diseases "Prof. Dr. Matei Balş" with the diagnosis: pansinusitis and intracranial suppurative complications.

The first girl: C.M.L., 14 years old, was admitted for fever, headache, vomiting and facial palsy. Clinically she evolved with rapid neurological deterioration, focal seizures, hemiparesis and then convulsive status. Brain MRI was suggestive for left brain abscess, subdural empyema and pansinusitis. With neurosurgical intervention and antibiotic therapy, the evolution was favorable, with full neurological recovery.

The second girl, V.E.S, 12 years old, was admitted in our hospital for frontal headache, fever, right palpebral edema, nausea and vomiting. In evolution she presented neurological complication with crural palsy and focal seizures. Brain MRI showed inter-hemispheric subdural empyema and suppurative pansinusitis. Otolaryngological surgical intervention and antibiotic therapy was administered, with favorable outcome, with no sequelae.

## Conclusion

Both girls had a good outcome but devastating short term and long term sequelae may occur without an early diagnosis and appropriate treatment. Brain MRI remains the gold standard for diagnosing any sinogenic intracranial complications. Both surgical and antibiotic treatment are essential for favorable evolution of suppurative intracranial complication in sinusitis.

## Consent

Written informed consent was obtained from the parents for publication of this Case report and any accompanying images. A copy of the written consent is available for review by the Editor of this journal.

